# Urotensin-II gene rs228648 polymorphism associated with the risk of diabetes mellitus

**DOI:** 10.1042/BSR20181275

**Published:** 2018-12-07

**Authors:** Yawei Zhao, Shuhua Fang, Kerong Que, Guangli Xu, Heng Zhang, Cong Qi, Nian Yang

**Affiliations:** 1 Department of Pharmacy, Jurong Hospital affiliated to Jiangsu University, Jurong 212400, Jiangsu, China; 2 Zhenjiang First People’s Hospital, Zhenjiang 212000, Jiangsu, China

**Keywords:** Diabetes mellitus, Meta-analysis, rs228648 polymorphism, Risk, Urotensin-II

## Abstract

**Background**: Urotensin-II (UII) rs228648 polymorphism has been reported to be associated with the risk of diabetes mellitus (DM) with inconsistent results. The present study sought to reassess the relationship between this polymorphism and susceptibility to DM by meta-analysis.

**Methods**: Relevant eligible studies and whole genome association study (GWAS) data electronically searched were pooled to evaluate the strength of the association with odds ratios (ORs) and 95% confidence intervals (CIs).

**Results**: Seven case–control studies involving 894 cases and 1186 controls were finally included in the meta-analysis. Overall analyses indicated that UII gene rs228648 variant was significantly associated with reduced risk of DM (allele, A vs. G: OR = 0.68, 95%CI = 0.56–0.82; dominant, AA+GA vs. GG: OR = 0.70, 95%CI = 0.53–0.91; homozygote, AA vs. GG: OR = 0.41, 95%CI = 0.28–0.61; recessive, AA vs. GA+GG: OR = 0.36, 95%CI = 0.19–0.71). In subgroup analyses based on ethnicity, the results showed a significant association of rs228648 polymorphism with decreased risk of DM in Chinese population under all five genetic models as well as in non-Chinese population under heterozygote and recessive models. Stratified analyses by specific type of DM also presented a significant association for common diabetes mellitus (CDM) under allele and homozygote as well as gestational diabetes mellitus (GDM) under all genetic models except for homozygote model. However, the synthetic analysis with GWAS data suggested an increased risk of DM with rs228648 effect allele in European population (OR = 1.01, 95%CI = 1.00–1.02).

**Conclusion**: The present meta-analysis preliminarily suggested a potentially opposite role of rs228648 polymorphism associated with DM risk in the Chinese and European population. Further studies are in great request to verify the results.

## Introduction

Diabetes mellitus (DM) posting one of the most common chronic diseases develops generally in individuals with insulin resistance in insulin target tissues and impaired insulin secretion from pancreatic β-cells in the presence of appropriate genetic and environmental factors [1,2]. Recently, a growing body of evidence proposes that a neurohormonal peptide named Urotensin-II (UII) may be implicated in the pathogenesis of DM [3].

UII initially isolated from the urophysis of teleost fish is a somatostatin-like cyclic undecapeptide and known as the most potent mammalian vasoconstrictor specified as yet [4,5], exerting its biological effects through interaction with its specific receptor that a member of a G-protein-coupled receptor superfamily, originally termed GPR14 [6–8]. It has been documented that one of the regions localized to 1p36-p32 in the UII gene showed potential linkage with type 2 diabetes (T2D) [9,10]. Elevated plasma levels of UII and increased UII expression have been also evidenced in patients with DM and in numerous diabetic tissues in previous studies [11–13]. Furthermore, it was reported that UII could reduce insulin secretion in response to glucose and arginine in the perfused rat pancreas, thereby, potentially contributing to a defective insulin secretion and/or aggravated insulin resistance in DM [14,15]. However, the underlying mechanism in details remains not elucidated yet.

Considering the potential influence of genetic alteration on the expression or activity of corresponding genes which may result in altered risk of diseases, a number of studies have investigated the role of polymorphic variations in UII gene in the susceptibility to DM to date. Expectedly, a previously identified single nucleotide polymorphism 143G>A (Thr21Met, rs228648) mapped on the exon 1 of UII gene has been reportedly linked to DM [16], however, results from relevant association studies are inconsistent. For instance, Okumus et al. [16] and Zhu et al. [17] found a significant association of UII rs228648 variant with a decreased risk of DM in the Turkish and Chinese populations, respectively, but Suzuki et al. [18] failed to show such association in the Japanese population. The discrepancy among previous studies may be due to the limited sample size or ethnicity variation. Therefore, to derive a more precise estimation, we performed a meta-analysis of available relevant studies to systematically reassess the association between UII gene rs228648 polymorphism and susceptibility to DM. Moreover, with regard to the potential counterpart in clinical characteristics and genetic context involved in the pathogenesis of DM [19–21], the studies conducted with the gestational diabetes mellitus (GDM) patients were also enrolled for the synthesis analysis regarding this association.

## Methods

### Literature search strategies

Potential eligible studies evaluating the association between UII gene rs228648 polymorphism and susceptibility to DM were electronically searched in Pubmed, Embase, Cochrane Library, Wanfang, China National Knowledge Infrastructure, and the Chinese Biomedicine Databases up to July 2018 using the following limits: Humans, and article in English or Chinese. We developed a search strategy using the following query in various combinations: [‘Urotensin II’ or ‘Urotensin-II’ or ‘UII’ or ‘U-II’] and [‘SNP’ or ‘variant’ or ‘polymorphisms’ or ‘polymorphism’]. Additionally, all the relevant references of the identified studies were further reviewed for additional eligible studies. If necessary, we contacted authors for additional data. In case of duplication, only the study with large sample size and complete information was selected.

### Selection criteria and data extraction

Studies would be considered eligible if they met the following criteria: (1) Studies assessing the association of UII gene rs228648polymorphism with the risk of DM, including GDM. (2) Studies with case–control design. (3) Studies containing available genotype frequency in cases and controls. (4) Studies providing sufficient information to estimate an odds ratio (OR) with 95% confidence interval (CI). The major reasons for exclusion were as follows: (1) Studies reporting duplicate data. (2) Studies without control design. (3) Studies lack of sufficient data to extract the genotype frequency or estimate the concerned ORs and 95%CIs. Information extracted from each individual study was collected by two independent reviewers using a uniform tabulation and any discrepancy was resolved by discussion or a consultation with a third reviewer to reach a consensus. The information was extracted as follows: first author, year of publication, country, ethnicity, types of DM, distribution of genotype or allele in cases and controls, source of control, genotype method, and evidence of Hardy–Weinberg equilibrium (HWE).

### Synthetic analysis of whole genome association study

Considering the limited sample size and population representation of the original case–control study, the additional synthetic analysis with whole genome association study (GWAS) study was also performed. GWAS data were retrieved with the T2D Knowledge Portal database (http://www.type2diabetesgenetics.org/). The ORs with 95%CIs of individual studies were extracted and synthesized to evaluate the association of rs228648 polymorphism effect allele with DM risk by Stata 12.0 software (Stata Corp., College Station, U.S.A.).

### Statistical analysis

Individual or pooled ORs with 95% CIs were calculated to assess the strength of associations between the UII gene rs228648 polymorphism and the risk of DM using Review Manager version 5.2 software (provided by the Cochrane Collaboration, Oxford, U.K.; http://www.cochrane.org/software/revman.htm). The significance of the pooled OR was determined by the *Z*-test and *P*<0.05 was considered significant [22]. The pooled analyses were performed with an appropriate effect model according to the between-study heterogeneity detected by the Cochran’s *Q-*test and the *I*^2^-test (*P*<0.10 and *I*^2^ > 50% indicated evidence of heterogeneity) [23]. In the presence of substantial between-study heterogeneity, the random effect model was used for the synthesis analysis; otherwise the fixed effect model was selected [24,25]. Sensitivity analysis was carried out by sequential omission of individual studies to check the stability of the results. Additionally, the potential publication bias was estimated by Begg’s funnel plot and Egger’s regression test by Stata 12.0 software (Stata Corp., College Station, U.S.A.) and *P*>0.05 indicated no evidence of publication bias [26,27].

## Result

### Study characteristics

**Figure 1:**
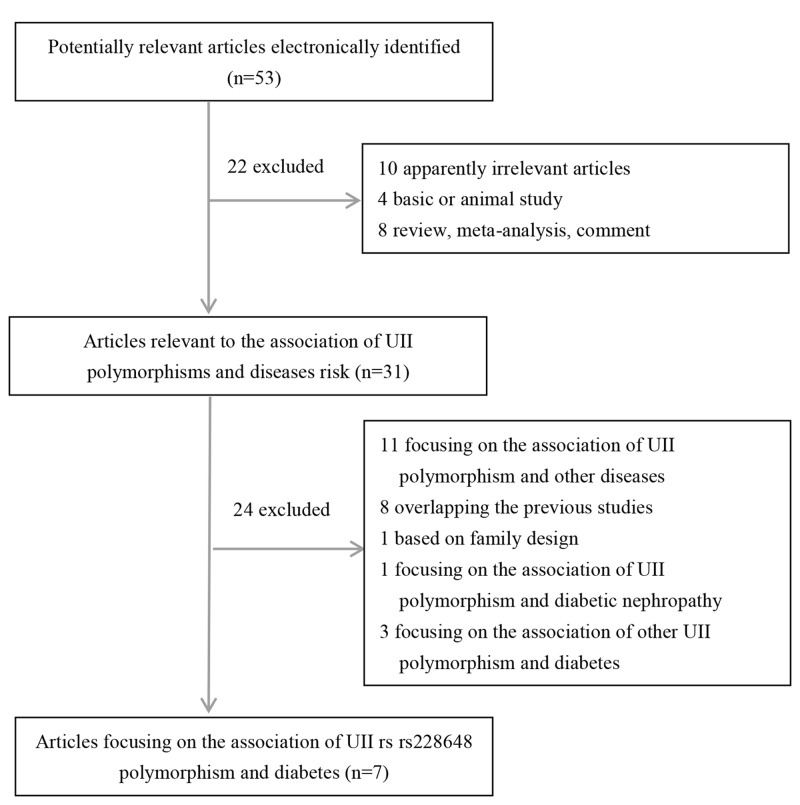
Flow chart of study selection

The flow chart summarizing study selection is shown in Figure 1. Totally, 53 potentially relevant studies were initially identified according to the search strategy. Applying the study selection criteria, seven case–control studies involving 894 cases and 1186 controls were finally included in the meta-analysis [16–18,28–31]. Of which, there were five studies conducted in Chinese population, and two studies conducted in Japanese and Turkish population, respectively, which were categorized as ‘non-Chinese’ population. In the included studies, five studies were examined for common DM (CDM) and two studies focused on GDM. Five studies were reported in Chinese and two were reported in English. The genotype frequencies of controls in each included study were in agreement with HWE. Detailed characteristics of selected studies are presented in Table 1.

**Table 1:** Characteristics of the included studies in the meta-analysis

First author	Year	Country	Ethnicity	Type	Genotype case	Genotype control	Source of control	Genotype method	HWE test
					AA/GA/GG	AA/GA/GG			
Suzuki*	2004	Japan	Japanese	CDM	109/195	83/161	Population	PCR-RFLP	Y
Sun*	2002	China	Chinese	CDM	113/297	111/205	Population	SBE	Y
Zhou	2015	China	Chinese	GDM	15/63/42	28/50/22	Hospital	PCR-RFLP	Y
Li	2011	China	Chinese	CDM	10/29/42	38/141/131	Hospital	PCR-LDR	Y
Tan	2006	China	Chinese	GDM	8/25/37	18/23/29	Hospital	PCR-RFLP	Y
Zhu	2002	China	Chinese	CDM	17/91/58	28/64/29	Hospital	PCR-RFLP	Y
Okumus	2012	Turkey	Turkish	CDM	10/99/4	127/138/26	Population	PCR-RFLP	Y

Abbreviations: CDM, common diabetes mellitus; GDM, gestational diabetes mellitus; HWE, Hardy–Weinberg equilibrium. Y, in agreement with HWE. *Only the allele distribution in cases and controls were provided in the two studies (A/G).

### Meta-analysis results

**Figure 2:**
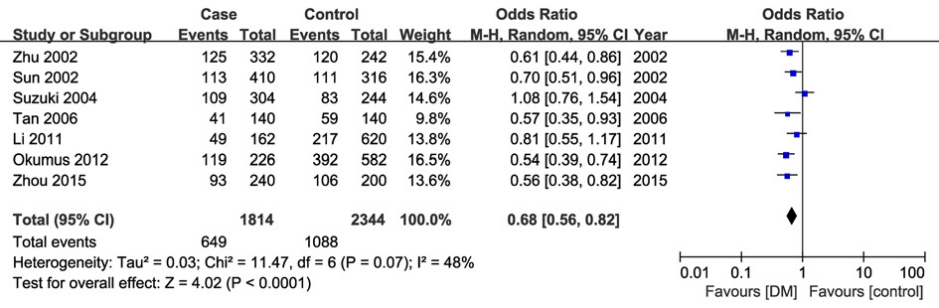
Forest plot for UII gene rs228648 polymorphism and DM risk under allele genetic model (A vs. G)

As shown in Figure 2 and Table 2, the overall results showed a significant association of rs228648 variant with reduced risk of DM under the allele genetic model when all the seven studies were pooled into the meta-analysis (A vs. G: OR = 0.68, 95%CI = 0.56–0.82). Since only the allele frequency was provided in the studies by Sun et al. [28] and Suzuki et al. [18], only other five studies were included in the pooled analyses under other four genetic models. Similarly, a significant association was observed between this variant and DM (dominant model, AA+GA vs. GG: OR = 0.70, 95%CI = 0.53–0.91; homozygote model, AA vs. GG: OR = 0.41, 95%CI = 0.28–0.61; recessive model, AA vs. GA+GG: OR = 0.36, 95%CI = 0.19–0.71).

**Table 2:** Meta-analysis results of the association between UII gene rs228648 polymorphism and DM risk

Contrasts	Number of studies	OR [95%CI]	*P*	Heterogeneity		Effect model
				*P*_h_	*I*^2^	
AA+GA vs. GG	5	0.70 [0.53, 0.91]	**0.007**	0.12	45%	F
Chinese	4	0.61 [0.46, 0.81]	**0.0006**	0.91	0%	F
Non-Chinese	1	2.67 [0.91, 7.84]	0.07	–	–	–
CDM	3	0.87 [0.45, 1.68]	0.67	0.04	69%	R
GDM	2	0.58 [0.37, 0.91]	**0.02**	0.59	0%	F
A vs. G	7	0.68 [0.56, 0.82]	**<0.001**	0.07	48%	R
Chinese	5	0.65 [0.56, 0.77]	**<0.001**	0.65	0%	F
Non-Chinese	2	0.76 [0.38, 1.51]	0.43	0.004	88%	R
CDM	5	0.72 [0.57, 0.91]	**0.006**	0.05	58%	R
GDM	2	0.56 [0.42, 0.76]	**0.0002**	0.97	0%	F
AA vs. GG	5	0.41 [0.28, 0.61]	**<0.001**	0.31	16%	F
Chinese	4	0.40 [0.27, 0.61]	**<0.001**	0.2	35%	F
Non-Chinese	1	0.51 [0.15, 1.76]	0.29	–	–	–
CDM	3	0.50 [0.30, 0.82]	**0.006**	0.2	39%	F
GDM	2	0.31 [0.17, 0.57]	**0.0002**	0.74	0%	F
GA vs. GG	5	0.90 [0.54, 1.51]	0.7	0.02	65%	R
Chinese	4	0.70 [0.52, 0.94]	**0.02**	0.94	0%	F
Non-Chinese	1	4.66 [1.58, 13.78]	**0.005**	–	–	–
CDM	3	1.13 [0.45, 2.83]	0.8	0.004	82%	R
GDM	2	0.73 [0.45, 1.19]	0.21	0.61	0%	F
AA vs. GA+GG	5	0.36 [0.19, 0.71]	**0.003**	0.002	76%	R
Chinese	4	0.48 [0.33, 0.69]	**<0.001**	0.16	42%	F
Non-Chinese	1	0.13 [0.06, 0.25]	**<0.001**	–	–	–
CDM	3	0.36 [0.11, 1.15]	0.09	<0.001	88%	R
GDM	2	0.37 [0.21, 0.64]	**0.0004**	0.98	0%	F

Values in bold indicate a significant association. Abbreviations: CDM, common diabetes mellitus; GDM, gestational diabetes mellitus; OR, odd ratio; 95%CI, 95% confidence interval; F, fixed model; R, random model; *P*_h_, *P*-value for the heterogeneity test.

In the subgroup analyses based on ethnicity, we also found a significant association in Chinese population (dominant model, AA+GA vs. GG: OR = 0.61, 95%CI = 0.46–0.81; allele model, A vs. G: OR = 0.65, 95%CI = 0.56–0.77; homozygote model, AA vs. GG: OR = 0.40, 95%CI = 0.27–0.61; heterozygote model, GA vs. AA: OR = 0.70, 95%CI = 0.52–0.94; recessive model, AA vs. GA+GG: OR = 0.48, 95%CI = 0.33–0.69) as well as in non-Chinese population (heterozygote model, GA vs. AA: OR = 4.66, 95%CI = 1.58–13.78; recessive model, AA vs. GA+GG: OR = 0.13, 95%CI = 0.06–0.25), however only one single study for the non-Chinese population was included. The stratified analyses by specific type of DM indicated a significant association of rs228648 polymorphism with decreased risk of CDM (allele model, A vs. G: OR = 0.72, 95%CI = 0.57–0.91; homozygote model, AA vs. GG: OR = 0.50, 95%CI = 0.30–0.82). Likewise, such association was also observed for GDM (dominant model, AA+GA vs. GG: OR = 0.58, 95%CI = 0.37–0.91; allele model, A vs. G: OR = 0.56, 95%CI = 0.42–0.76; homozygote model, AA vs. GG: OR = 0.31, 95%CI = 0.17–0.57; recessive model, AA vs. GA+GG: OR = 0.37, 95%CI = 0.21–0.64).

### Publication bias and sensitivity analysis

**Figure 3:**
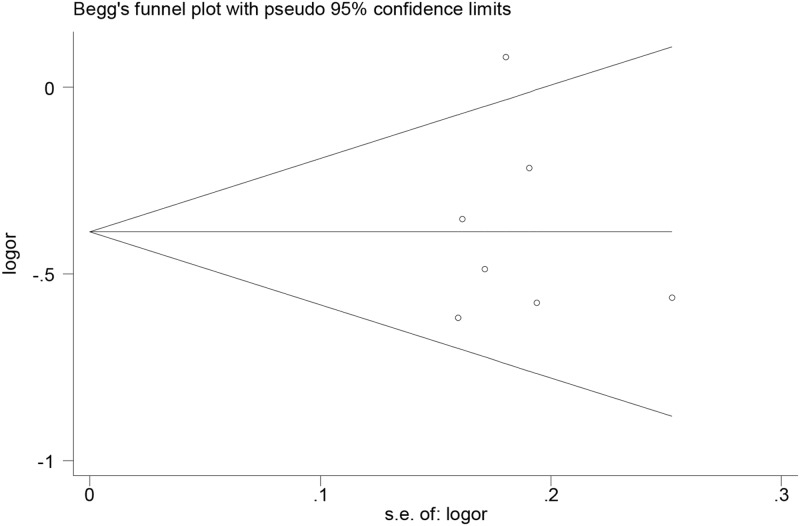
Funnel plot for UII gene rs228648 polymorphism and DM risk under allele genetic model (A vs. G)

As shown in Figure 3, the Begg’s funnel plot for publication bias test showed no obvious asymmetry, indicating no evidence of publication bias, which was further supported by the Egger’s test (A vs. G: *P*=0.958>0.05). For the sensitivity analyses by sequential omission of individual studies, no substantial alteration for the pooled ORs was observed under the allele, homozygote and recessive models; however, the significance of pooled ORs was excessively affected by excluding the study by Zhu et al. [17] and Zhou et al. [29] under dominant model and excluding the study by Okumus et al. [16] under heterozygote model. Nevertheless, most results of the sensitivity analyses still revealed a significant association, suggesting the reliability and robustness of the meta-analysis.

### Synthetic analysis of GWAS studies

**Figure 4:**
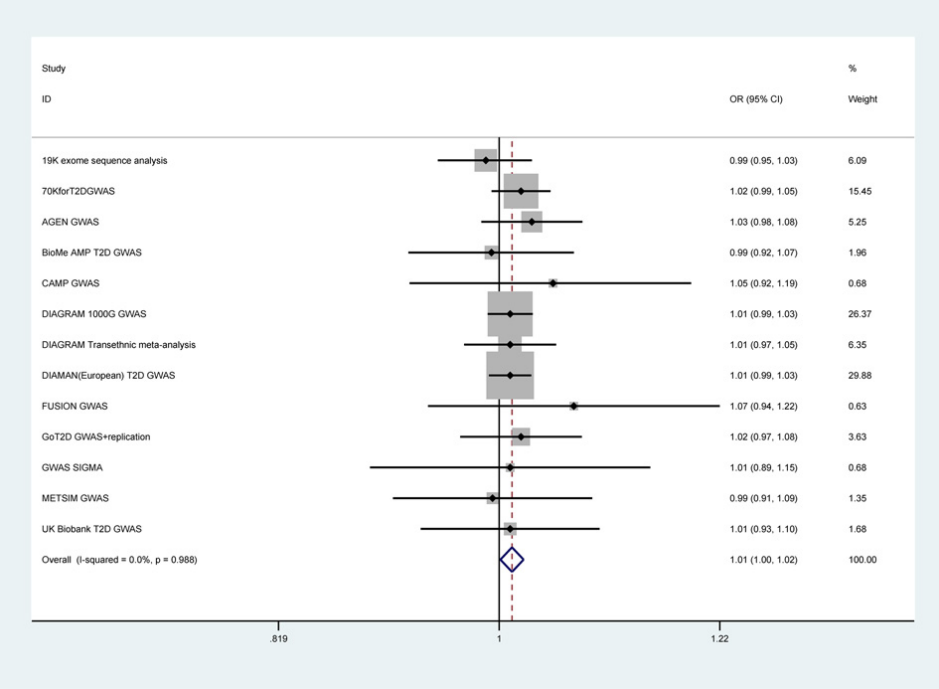
Forest plot for the effect allele of rs228648 and DM risk with GWAS data (A vs. G)

As shown in Figure 4, a total of 13 GWAS studies were retrieved after excluding the GoT2D GWAS dataset which was duplicated from the GoT2D GWAS+replication dataset with a larger sample size. All the GWAS studies were mainly conducted in European population. As a result, the synthetic analysis with a fixed effect model showed a significant association of rs228648 polymorphism effect allele with increased DM risk (OR = 1.01, 95%CI = 1.00–1.02).

## Discussion

The study is the first to reliably quantify the association between UII gene rs228648 polymorphism and susceptibility to DM. Our meta-analysis implied a potentially opposite role of rs228648 polymorphism in different populations, suggesting that the UII rs228648 variant seemed to be associated with a reduced risk of DM in the Chinese population while an increased risk in European population.

Over decades, numerous polymorphic regions in UII gene encoding for UII peptide, including rs228648 polymorphism, have been identified and correlated to many diseases, such as DM, diabetic retinopathy [16], breast cancer [32], Behcet’s disease [33], and systemic sclerosis [34]. Notably, Yumrutas et al. [32] found that Thr21Met (143G>A, rs228648) polymorphism in the UII gene was associated with the risk of developing breast cancer and the variant genotype was significantly associated with reduced plasma expression of UII, proposing a possible mechanism for UII involvement in the altered risks of diseases. Furthermore, Zhou et al. [29] found that the variant AA genotype was associated with a decreased risk of DM and impaired insulin resistance in patients with GDM. Additionally, the study by Ong et al. [35] revealed that the GGT haplotype (−605G, 143G, and 3836T) was associated with higher plasma level of U-II, insulin and higher homeostasis model assessment of insulin resistance index and β-cell function, suggesting the possible role of UII 143G>A (rs228648) polymorphism in the glycemic control via the regulation of plasma UII levels and insulin resistance. Although relevant studies have prompted a potential genetic association of UII gene rs228648 polymorphism with DM, the results seem to be inconsistent; the underlying mechanism for the association of this polymorphism with DM remains largely unknown as well.

Overall, the present meta-analysis involving 894 cases and 1186 controls from seven case–control studies suggested a significant effect of UII gene rs228648 variant on the decreased risk of DM. In the subgroup analysis based on ethnicity, we found a significant association between UII gene rs228648 polymorphism and reduced risk of DM in Chinese population under all five genetic models with relatively less heterogeneity but only under heterozygote and recessive models, suggesting that this association may be comparatively evident in Chinese population. However, only few studies were included in the analysis regarding non-Chinese population, more necessity is required for validation of this association in other populations. In addition, the stratification analysis by specific types of DM indicated an association of UII gene rs228648 polymorphism with CDM as well as GDM, suggesting a possibility that the UII gene rs228648 polymorphism may causally and partially contribute to the pathogenesis of CDM and GDM on the basis of similar hereditary context [20,21]. However, this association was potentially evident in GDM due to more significant associations suggested by more models with less between-study heterogeneity in the present study. Whereas, this findings should be interpreted with cautions because the studies concerning the association between the variant and DM are all conducted in Chinese population and with limited sample size. Conversely, a significant association of rs228648 effect allele with increased DM risk was indicated in European population, suggesting the potential importance of ethnicity difference concerning the association. Additionally, the gene–gene or gene–environment interaction, and the relation between marker and causal variants may also contribute to the difference. Nevertheless, several concerns should also be noted for the GWAS studies conducted in European population in consideration of only approximately 1% increase in DM risk for the effect allele, the lack of detailed data on genotype distribution in cases and controls, and the potential population stratification. Accordingly, more studies with well design are still needed to verify our results and explore the underlying mechanism.

Despite an appropriate approach applied for the present study, some limitations should be acknowledged yet. First, most of the included individual studies were retrieved from Chinese population, however, the rs228648 polymorphism is most predominant in the South Asian and European populations compared with East Asians. No population from South Asian, American, and fewer population from other East Asian countries were represented in the present study, thus our results may be primarily applicable to limited populations. Second, limited studies conducted in the Chinese population were enrolled in the meta-analysis in spite of no evident publication bias indicated, which would limit our confidence in drawing conclusions especially in the subgroup analysis regarding the specific forms of DM and ethnicity. Third, our estimates were based on unadjusted data, some important compounding factors such as BMI, gender, age, etc., were not taken into consideration due to the lack of original information. Fourth, as DM is multifactor-affected disease, potential environment–gene, gene–gene interactions including haplotype effects may be of importance for the observed disease-effect unconformity [16], but we had insufficient data to perform an evaluation of such interactions.

## Conclusion

In conclusion, the present meta-analysis suggested that UII gene rs228648 polymorphism may be associated with a decreased risk of DM in Chinese population but an increased risk in European population. In light of the limitations existed, additional larger studies allowing stratification analyses for environment–gene interaction, haplotype effects, type and ethnicity specifications are greatly suggested to further clarify the possible role of the concerned genetic variant in the etiology of DM in future work.

## Abbreviations

CDM: common diabetes mellitus
CI: confidence interval
DM: diabetes mellitus
GDM: gestational diabetes mellitus
GWAS: whole genome association study
HWE: Hardy–Weinberg equilibrium
OR: odds ratio
T2D: type 2 diabetes
UII: Urotensin-II
